# Industrial-Graded Epoxy Nanocomposites with Mechanically Dispersed Multi-Walled Carbon Nanotubes: Static and Damping Properties

**DOI:** 10.3390/ma10101222

**Published:** 2017-10-24

**Authors:** Andrea Giovannelli, Dario Di Maio, Fabrizio Scarpa

**Affiliations:** 1 Nano-Tech Società per Azioni, Zona Industriale Campolungo, 105 63100 Ascoli Piceno (I), Italy; info@italnanotech.com; 2 Bristol Composites Institute (ACCIS), University of Bristol, Bristol BS8 1TR, UK; Dario.DiMaio@bristol.ac.uk

**Keywords:** carbon nanotubes, epoxy, mechanical characterization, dispersion, damping

## Abstract

The majority of currently published dispersion protocols of carbon nanotubes rely on techniques that are not scalable to an industrial level. This work shows how to obtain polymer nanocomposites with good mechanical characteristics using multi-walled carbon nanotubes epoxy resins obtained by mechanical mixing only. The mechanical dispersion method illustrated in this work is easily scalable to industrial level. The high shearing force due to the complex field of motion produces a good and reproducible carbon nanotube dispersion. We have tested an industrial epoxy matrix with good baseline mechanical characteristics at different carbon nanotube weight loads. ASTM-derived tensile and compressive tests show an increment in both Young’s modulus and compressive strength compared with the pristine resin from a starting low wt %. Comparative vibration tests show improvement in the damping capacity. The new carbon nanotube enhanced epoxy resin has superior mechanical proprieties compared to the market average competitor, and is among the top products in the bi-components epoxy resins market. The new dispersion method shows significant potential for the industrial use of CNTs in epoxy matrices.

## 1. Introduction

Composites materials are made from two or more constituents, with generally different physical or chemical properties. The combination of these phases produces a material with characteristics that differ from the isolated components. In the field of fiber-reinforced polymers (i.e., glass fiber composites, carbon fiber composites, Kevlar composites) the polymeric matrix is responsible for holding together the fiber portion of the material, and it is itself a structural element contributing to the final mechanical proprieties of the composite [1,2]. Despite this, the mechanical resistance of polymeric matrices is generally low compared to the fiber, and the failure of the composite is mainly due to matrix-fiber debonding or matrix micro-crack for general off-axis loading (CIT). It is therefore evident that any mechanical improvement of the polymeric matrix will increase the resistance of the final composite material. The need to obtain composites with peculiar properties and high specific stiffness/resistance has often pushed researchers to investigate the value of carbon nanotubes-based composites [3,4,5,6]. Since the modern re-discovery of carbon nanotubes (CNTs) in 1991 [7], an impressive body of literature has been published on the subject, especially regarding the properties of materials in which CNTs are used as additives [3,8,9]. Although CNT-based nanocomposites have been used quite extensively in industrial products for several years (e.g., Babolat tennis racquets, Baltic Yacht) [8,10], the main obstacles currently recognised as preventing greater industrial use of CNT-based nanocomposites are the relative high cost of CNTs, lack of good dispersion in a cost-effective manner [11] and the large increase in the viscosity of the resins following the dispersion of the CNTs [12]. While the use of cheaper industrial grade CNTs can circumvent the first problem, the remaining two provide a greater challenge to the widespread use of CNT-based composites. 

The degree of dispersion of the nanoparticles into the matrix has been shown to influence the characteristics of the final products [3,13,14]. The most commonly used method to disperse CNTs in polymer matrix is sonication, often accompanied by the use of diluent and surfactants [13,15]. Those methods are suitable to prepare small batches, and require time consuming and labour-intensive procedures. The dispersion by mechanical method is probably the only approach that can be used by industries for large-scale production [16]. The increase in matrix viscosity following the dispersion of CNTs can be partially avoided by adopting some methodological aspects (i.e., higher working temperatures, use of solvents, lower viscosity starting resins), but it will likely limit the use of high (>2–5 wt %) CNTs loading for industrial applications. The literature shows that relatively low strain-to-failure epoxies can improve toughening by adding nanoparticles [13,15,16,17]. The characteristics of the resulting CNTs-based epoxy are, however, below the market standards for commercial non nano-based epoxies (see Table 1). To evaluate the real potential of this CNT-based materials for industrial applications, we have used in this work a commercial epoxy matrix currently widely employed in industrial composite productions. We have applied a new dispersion protocol based only on mechanical dispersion to test the capacity of the new CNTs-based nano-composites, to be used in a simple and cost-effective way from an industrial perspective. Mechanical tensile and compressive tests have been carried out on epoxy and CFRP CNT-doped samples to verify the effect of the new dispersion methods over the properties of the nanocomposites. Vibration tests have also been performed on the CFRP-based nanocomposites for a potential evaluation of the dynamic properties in full-scale composite layouts. All results have been postprocessed using a robust statistical technique to assess the significance of the data versus the level of the CNT dispersions.

## 2. Materials, Manufacturing and Experimental Methods

### 2.1. Epoxy and CNTs

**Figure 1:**
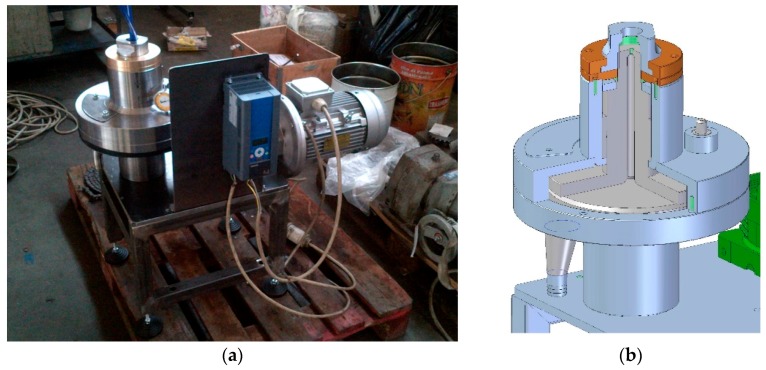
View of the CNT dispersion apparatus (**a**) and its internal schematics (**b**).

The epoxy matrix used is in this work is a EC 157 bi-component epoxy resin (Elantas Camattini, Collecchio, Italy) cured with an amino-based slow curing agent (XLR curing agent, Elantas Camattini) with a 100:30 epoxy/curing agent weight ratio. EC 157 is a transparent and low viscosity resin with good mechanical properties, and a low temperature curing cycle. We used non-functionalized multi-walled carbon nanotubes (MWCNTs) with an average aspect ratio of 160% and 90% purity (Nanocyl s.a., Sambreville, Belgium), average diameter *d* = 8 nm and length *l* up to 1.5 μm. The epoxy resin was loaded with 0.1, 0.2 and 0.5 parts of CNTs per hundred resin (phr), and with adding the curing agent the final weight load was 0.0769%, 0.1539% and 0.3846% respectively. The MWCNTs were dispersed in the epoxy resin with a patented dispersion method that uses high shear force (patent request n. MO2013A000303, see Figure 1). The fluid is forced to pass through different surfaces in relative motion with a gap between them from 5 mm to 5 μm, creating therefore a complex 3D motion field (I, Figure 1b). The nano-based epoxy obtained in this way was then mixed with the curing agent (100:30 ratio) and degassed under vacuum for 20 min. The epoxy specimens were cast in a silicone mould and cured for 10 h at 35 °C and followed by 7 h at 70 °C with a ramp of 1 °C/min (ramp-up and ramp down for cooling). The same process was applied to all the weight loading cases considered in this work. To make a non-CNT doped control sample, the process was also applied to pristine epoxy resin specimens. The samples were subjected to tensile and compressive tests in accordance to ASTM D 638 and ASTM D 695 M standards. Strain data from full-field analysis were measured using a Dantec’ Digital Image Correlation DIC-Q400 system with a 5 MPixel camera (Dantech Dynamics, Skovlunde, Denmark). The surface of the specimens was smoothed to have booth surface parallel and marked with white dots using acrylic paint to aid the strain measurement. The samples were characterized using SEM on the surfaces of fractured specimens. Atomic force microscopy (AFM, NT-MDT Ltd., ETALON probe series, Moscow, Russia) was also used. The overall size of the chip used for the scanning was 3.6 × 3.6 × 0.45 mm^3^, with images taken with one pass using a semicontact error scanning mode technique (silicon tip of 9 microns, cone angle <= 22° and curvature radius of 10 nm). The nanocomposite chip was put on an Au substrate. Further characterization was also performed using Dynamic Light Scattering (DLS) techniques at 25 °C on samples with 0.1% and 0.2% CNT immediately after manufacturing, and 33 and 125 days from the production. The DLS was performed using a DLS Zetasizer Nano S, Malvern Instruments Ltd. (Malvern, Worcestershire, UK). A He–Ne laser with a power of 22 mW emitting polarized light at a wavelength of 632.8 nm was used as the incident beam. The DLS measurements were performed at 25 °C with an acquisition time of 60 s. The scattering angle was 90°.

**Figure 2:**
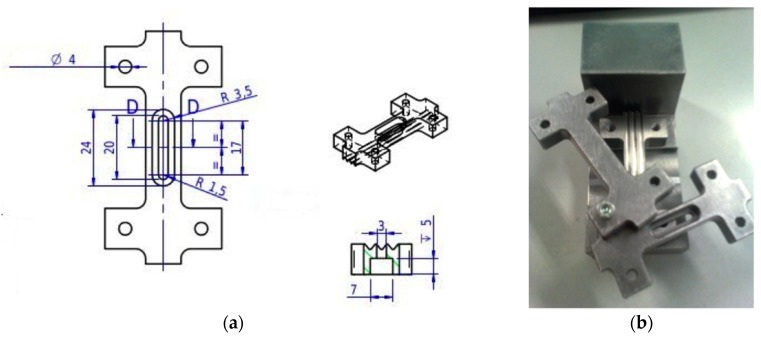
CAD design (**a**) and image (**b**) of the modified jig for compression tests.

The samples were also subjected to compressive tests carried out in accordance with ASTM D 695 M using a jig for thin specimens with a Zwich Roell machine with a 50 kN load cell (Zwick GmbH & Co, Ulm, Germany) and a testing speed of 1.3 mm/min. The sensibility of the load cell was tested with a static calibration. The compressive tests were performed with pristine resin (0 phr), 0.1, 0.2, and a further batch of 0.5 phr samples. It is normal practice to evaluate the compressive strength and the compressive modulus performing two different types of tests. The ASTM D 695 is suitable to obtain the ultimate compressive strength, but the strain gauges are not easy to apply (in particular for thin specimens) because of the particular jig required by the standard, moreover the video gauge is not suitable to be used in this particular testing configuration. It is therefore necessary to carry out a second test following the ASTM D 6441 protocol to obtain the compressive modulus. In this work the characteristics of the material were obtained using the ASTM D 695 M standard by building a modified jig with a buttonhole suitable to the use of video gauge (Figure 2). The buttonhole was designed not to affect the geometry of the grinded surface and the distance and number of teeth required. This method is inspired to the ICSTM fixture design [18,19].

### 2.2. CFRP Samples

The CFRP panels were prepared using a wet layup approach. The carbon used is a TC33 Tairyfill plain woven at 200 g/m^2^. The epoxy resin was mixed with the curing agent (100:30 ratio) and weighted to reach a fiber volume fraction of 50%. Each ply was impregnated and then 6 plies positioned on an aluminium plate. Vacuum was applied and the laminate cured in autoclave at 6 bars for 10 h at 35 °C, followed by 7 h at 70 °C with ramp of 1 °C/min for both temperature increase and cooling–similarly to the procedure applied to the epoxy matrix samples.

Vibration tests have been carried out on these samples using an electromagnetic shaker (maximum drop out force of 50 N) and a laser Doppler Vibrometer (Polytec PSV-400, Polytec GmbH, Waldbronn, Germany). The samples were attached to the shaker in a central position through a support connected to a PCB force transducer. The excitation position and the measurement grid were kept constant for all the samples tested. Four specimens for each CNTs weight load were tested, and their first three vibration modes excited and measured. Each mode was measured using 40 Fast Fourier Transforms (FFT) lines around the resonance peak. The data from the scanning laser vibrometer were imported into ICATS modal analysis software (Rel. 2.0, Imperial College, London, UK) [20] for post-processing. Each single resonance curve was curve-fit with a Line-Fit algorithm [21], and the modal damping ratios and natural frequencies for the three modes calculated.

### 2.3. Data Analysis

Five specimens for each test were used, except for the traction test related to the 0.2 phr load because of a premature failure in one of them. The measured variables were the tensile stress (*σ*), tensile modulus (*E*), compressive stress, compressive modulus, the elongation at yield point (*ε*_YP_) and the elongation at break (*ε*_b_). The data recorded by the test machines and by the video gauge were analysed and the *σ* − *ε* correlation performed. The data were tested about the normality of the distribution using the Pearson coefficient [21]. Outliers were determined in accordance with ASTM E178–02 using the test criterion *Tn* with 0.5% upper significance level:

*Tn* = (*M* – *X*)/*S* (1)

where *M* is the average, *x* the value of the measure and *s* the standard deviation of the population.

Seven outliers were identified out of a population of 28 values. After the removal of the outliers the Pearson coefficient showed a symmetrical distribution. A two-tailed t-student test was also carried out for every set of value, comparing each CNTs phr load set with the corresponding pristine resin configuration.

## 3. Results

**Figure 3:**
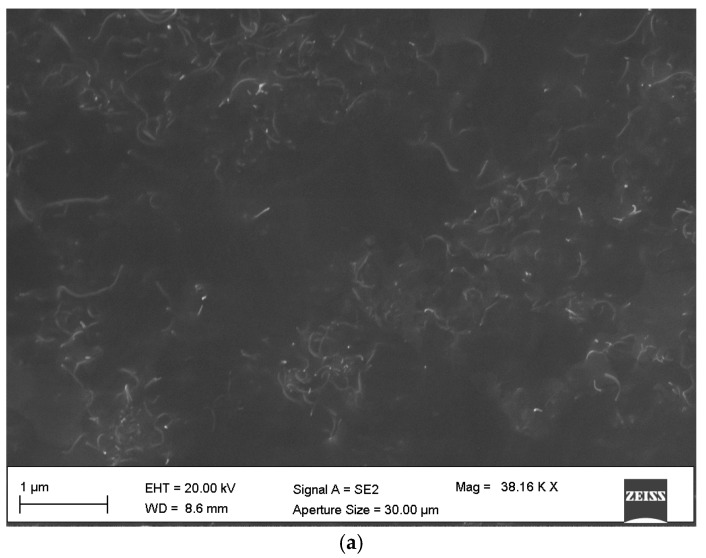

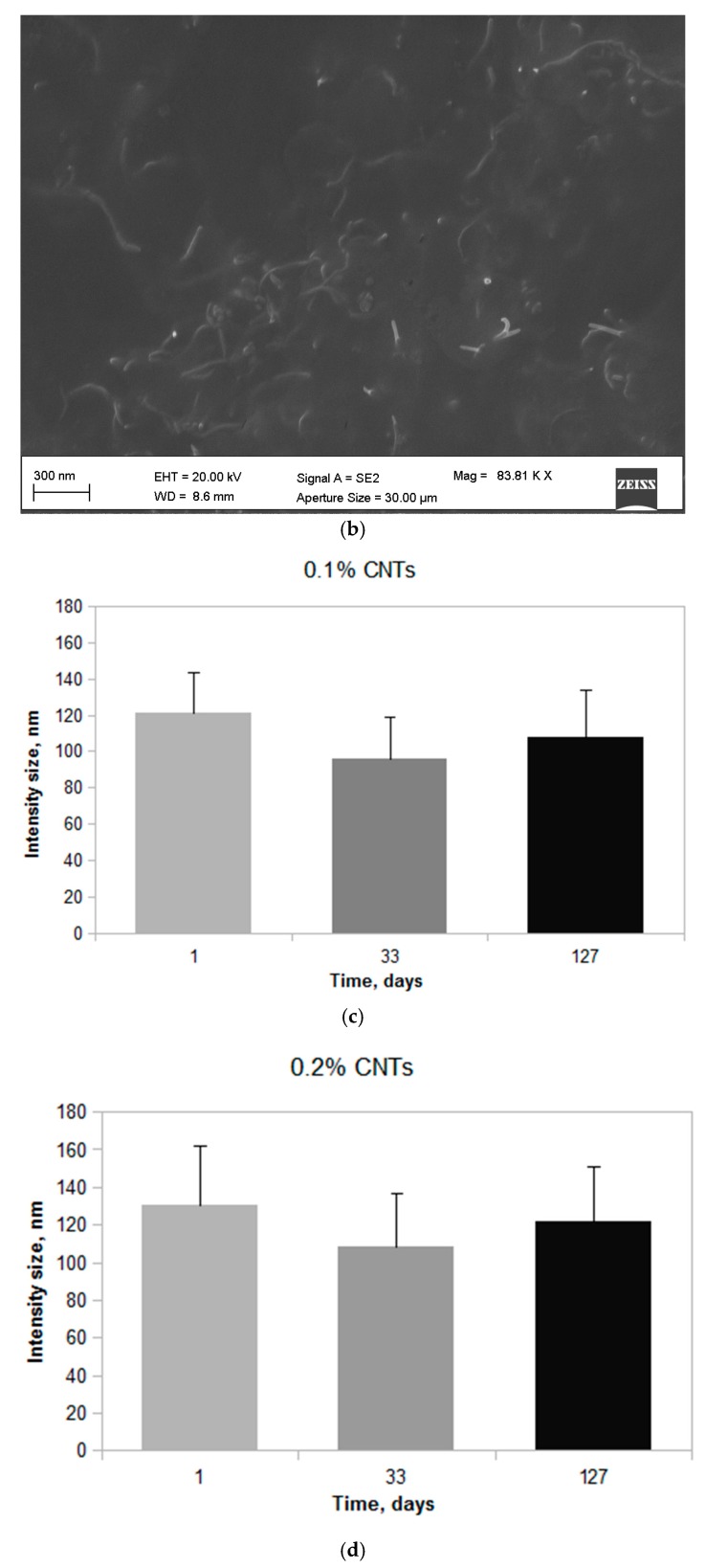
(**a**,**b**) SEM images of fracture (tensile) surfaces of CNTs epoxy samples (0.5 phr); (**c**,**d**) DLS measurements of the doped resin after different elapsed times for 0.1% and 0.2% phr, respectively.

The dispersion of the carbon nanotubes was investigated using a SEM analysis of specimens’ failure surfaces (Figure 3a,b). From the images it is apparent the presence of generally good and uniform distributions of the CNTs through the matrix. Some areas with more CNT densification are also present, but no significant bundles were observed. The images are related to a specific section within the composite. It must be noticed that the dispersion is in general three-dimensional, with inhomogeneity likely being present through the thickness of the composite [22]. It is possible however to observe the formation of a complex networked structure, with induced waviness and distortion of the nanotubes during the sonication process. We also carried out Dynamic Light Scattering (DLS) tests at 25 °C on samples with 0.1% and 0.2% CNT immediately after manufacturing, and 33 and 125 days from the production (Figure 3c,d). The data show a clear cluster of the probability density function of the intensity around the ~100 nm size for the CNTs hydrodynamic radius of the particles. This is far larger than the nominal diameter of the tubes, and clearly shows that the great majority of the particles had hydrodynamic dimensions between 80 nm and 200 nm. It is quite significant to observe that the PDF of the intensity did not change between the immediate production and a long period of time, showing that the dispersion technique used is robust and provides stable performance.

The results of the tensile tests are shown in Table 2. The tensile strength showed no significant difference between the pristine epoxy and both the CNT-based epoxies. While the elongation at the yield point did not vary significantly between the pristine epoxy and the 0.1 phr samples, the 0.2 phr specimen showed however a significant reduction (*t*-test, *p* < 0.013). Similarly, the elongation at the break increased significantly for the 0.2 phr (*t*-test, *p* < 0.002). The Young’s modulus improved in a remarkable way in the 0.2 phr epoxy with a 17.8% increase compared to the pristine resin (*t*-test, *p* < 0.03), reaching a maximum of 3.89 ± 0.20 GPa.

The results from the compressive tests are shown in Table 3. Contrary to the case of the tensile strength, the compressive tests showed a significant enhancement of the compressive behavior in the ultimate compressive strength (overall with 20%), with an increase from 94.86 ± 4.65 MPa (pristine resin) to 113.74 ± 3.38 MPa in the 0.5 phr epoxy. The compressive modulus shows an increment from the 0 phr to the 0.5 phr (*t*-test, *p* < 0.031). However, the elongation at yield point showed an initial decrease at 0.1 and 0.2 phr, followed by a 32% increase in the 0.5 phr. The elongation at break was not measured due to the particular design of the ASTM jig.

We have used the Halpin-Tsai model for planar, randomly oriented discontinuous fibers to calculate the tensile Young’s Modulus of the composites [23,24,25] to compare the experimental data with theoretical predictions:

*Ec* = *Em* {[3/8 (1 + 2(1/*d*) *η_L_V_NT_*/(1 – *η_L_V_NT_*)] + 5/8 [1 + 2 *η_L_V_NT_*]/ (1 – *η_L_V_NT_*)] } (2)

where:

*η_L_* = [(*E_NT_/E_m_*) – 1]/[(*E_NT_/E_m_*) + 2 (1/*d*)] (3)

*η_L_* = [(*E_NT_/E_m_*) – 1]/[(*E_NT_/E_m_*) + 2 (4)

In Equations (2)–(4), *E_c_* is the Young’s modulus of the composites, *E_m_* the one of the pristine epoxy and *E_NT_* the Young’s modulus of the carbon nanotubes. The average carbon nanotube length is *l*, while *d* is the average outer diameter of the carbon nanotubes and VNT the volume fraction of carbon nanotubes in the epoxy. For the CNTs we have adopted the theoretical Young’s modulus of 1 TPa [26,27]. The volume fraction of the CNTs was calculated using their weight fraction, density (2250 kg/m^3^), and the density of the epoxy (1150 kg/m^3^) plus the curing agent (950 kg/m^3^) for each weight load. The theoretical Young’s modulus, calculated for each weight load of CNTs, resulted in 3.40 and 3.50 GPa and was inferior to the experimental measured modulus of 6% for the 0.1% and 11% for the 0.2 phr epoxy.

Regarding the damping performance of the samples, from Table 4 it can be seen that there is no significant improvement in the loss factor for the first mode of vibration, while the second mode shows a very significant enhancement between the loss factor of the pristine panel and the 0.5 phr case by 489% (*t*-test, *p* < 0.0003). For the third mode the increase of the loss factor is 105% for the 0.1 phr (*t*-test, *p* < 0.006) and 110% for the 0.5 phr (*t*-test, *p* < 0.0053), but no significant improvement is observed in the 0.2 phr laminate. The inertia of the shaker could provide an added mass effect if we consider the low weight of the samples, but that factor would affect all specimens equally, and therefore could be considered systematic and negligible.

## 4. Discussion

In the literature, the value of the theoretical Young’s modulus considered in this work is the theoretical upper value under the assumption of perfect dispersion and impregnation of the CNTs [16], and in all published papers it is higher than the measured value. One possible explanation lies in the dispersion of CNTs through sonication, that which is likely to affect the final CNTs average length and damaging the nanotubes [28,29,30,31]. This factor may in turn influence the mechanical properties of the epoxy and justify the higher theoretical value obtained using the Halpin-Tsai equation, because of the use of higher diameters in the formula than the ones effectively present in the CNTs because of the sonication damage. Sonication can also disperse the CNTs aggregates with two different mechanisms: exfoliation and breaking of the carbon nanotubes. The former is not thought to be responsible for the damage of the CNTs, while the latter is dependent upon the time of exposure to the sonication. At the beginning of the sonication treatment there are only aggregate that can be exfoliated or broken into small bundles, but over time the percentage of free carbon nanotubes or small aggregate in the media that could be damaged by the ultrasonic waves increases significantly. As a result of this mechanism, a large number of very short and damaged CNTs will be present, in addition to a considerable amount (in weight) of aggregates that will affect the hypothesis of perfect dispersion and impregnation and the real average length of the CNTs adopted in the equation.

The production of CNT by Fluidized Bed Chemical Vapour Deposition brings large quantities of aggregates in the range of ten to hundreds of microns. These bundles consist of CNT, impurities and catalysts and are directly the result of van der Vaals interactions. Lattice defects, aspect ratio, and number of walls affect the moment of inertia of the CNTs, hence more flexible and longer CNT tends to form more complex bundles that will be more difficult to disperse [14]. Single-wall carbon nanotubes (SWCNTs) with large aspect ratios theoretically provide a higher specific surface area and better performance when added to a polymer matrix. However, their flexibility and their high aspect ratio imply also produce strong bundle formations that are difficult to disperse. As a final result, SWCNTs when sonicated are subjected either to a poor dispersion or to large breakage. The use of MWCNTs with significant moment of inertia values and short aspect ratios (i.e., large diameters and wall numbers) enables easier dispersion by sonication. Therefore, the widespread use of MWCNTs as fillers can be justified, also in part also due to their lower capital costs compared to SWCNTs.

A second aspect to consider when trying justifying differences in the literature about the performance of CNTs as fillers is the effective load of the carbon nanotube used. In published literature, the measured mechanical properties of CNT-based polymers are often referred to the CNTs weight load [13,14,16]. Due to the nature of carbon nanotubes [32], at equal weight loads the total number of CNTs can vary considerably depending on the number of walls, length and diameter of the tubes. This will affect the properties of the nanocomposite and significantly increase the difficulties in comparing different studies, and therefore we argue that the use of the weight loading is not a necessarily useful metrics to benchmark different nanocomposites with the same type of matrix.

Tensile strength shows no improvement compared to the value of the pristine matrix. A weakening in ultimate tensile strength through the use of CNTs is often reported in the literature [13] and it is usually attributed to the creation of defects and aggregates associated with difficulties in the preparation of the specimen. In this study we have used a relatively stiff matrix (3.301 ± 0.185 GPa), with an ultimate tensile strength of 73.96 ± 0.66 MPa. Ci and Bai have demonstrated that the contribution of the CNTs to the improvement of the tensile strength mainly depends on the stiffness of the epoxy [32].

Equations (2)–(4) are consistent with applications of classical elasticity. In nanomechanics it is, however, current thinking to use nonlocal elasticity theory *a’ la* Eringen to interpret discrepancies between the results provided by classical local elasticity and dispersions from experimental or molecular mechanics data [33]. The departures of the experimental data from the local elasticity formulas may also be partially explained by the use of nonlocal parameters in Eringen theory to consider the scale size effect of the nanolattice over the global mechanical properties of the nanostructure. We observe however that in nonlocal CNT models describing bending and other complex mechanical loading the variation of the Young’s modulus versus the nonlocal parameters tends to be confined around 26% at the best [32]. The larger experimental variations that we observe in this work and others in open literature seem more likely to be ascribed to the other physical phenomena that we are discussing in this work.

An important point is also associated to the relation between the critical length of the CNTs (*l_c_*), the CNTs ultimate tensile strength (*σ_CNT_*) and the interface strength (*τ_CNT_*) between CNTs and epoxy matrix [34]. By using the critical length formula referred to the average external diameter (*d_e_*) of the CNTs, and dividing by the length we can find the average critical aspect ratio (*AR_c_*):

*lc* = *σ_CNT_de*/2*τ_CNT_* → ARc = *σ_CNT_de*/2*τ_CNT_* (5)

In which *AR_c_* = *l_c_*/*d_e_*. The allowable range for *σ_CNT_* can be considered as 11–40 GPa < *σ_CNT_* < 50–63 GPa [25,26,35,36]. The interface strength *τ_CNT_* varies between 2 MPa and 128 MPa [26,36,37,38,39,40]. Using those values, the critical aspect ratio of the carbon nanotubes can vary between 42.97 and 5250, with an average of 2646—well above the average aspect ratio of the CNTs used in this work (160). Due to the high stiffness of the matrix and a CNT average aspect ratio well below the critical one, the preferred mechanism occurring during tensile deformation of the nanocomposites is the pull out of the CNTs, which contributes almost nothing to the enhancement of the ultimate tensile strength.

The ultimate compressive strength, however, is quite significantly improved, also suggesting that the CNT critical length is not a key parameter in compression loading. As indicated by Schadler et al. [40], the improvement in the compression performance can be explained by the fact that during the load transfer to the MWCNTs only the outer layers are stressed in tension, whereas all the layers respond to compression through easy buckling and bending of the nanotubes. In the same paper, Schadler et al. reported a greater increase in compression Young’s Modulus than during traction, but no data about tensile and compressive stresses are indicated on that paper, and no information about the exact morphology of CNTs are provided. Similarly to the tensile case, the stiffness of the epoxy plays an important role, and a minor stiffness of the epoxy in compression can further highlight the contribution made by the CNTs.

The present work has shown that the Young’s Modulus has been improved by the use of carbon nanotubes more under tensile loading than under compression, but the opposite occurred regarding the strength. It may be argued that the load transfer mechanisms outlined above are responsible both for compressive strength and Young’s Modulus improvement, but with different magnitudes depending on the CNTs used. The comparison between the mechanical properties provided by the large-scale dispersion technique used in this study and average values of commercially available nanocomposite epoxies with similar viscosity, glass transition temperature and curing cycle is quite favourable for our nanocomposites. We measure between 3.640 and 3.890 GPa and 74 and 72 MPa for the tensile Young’s modulus and strength, against average values of 2.783 GPa tensile Young’s modulus and 65 MPa of tensile strength found in open literature. No comparison for compressive behavior was possible due to not enough data available.

The CFRP panel fabricated using the MWCNTs-based epoxy showed a good increase in loss factor compared to the pristine with the 0.5 phr at certain frequencies. It is apparent that below a certain amount of CNTs the effect on the loss factor is negligible. Statistically significant increases of the modal loss factor are observed for the 3rd mode of the 0.1 and 0.5 phr. The CNTs dissipate energy when the vibration triggers stick-slip phenomena [11,40]. In particular, when the onset of slip between the carbon nanotubes and the matrix is triggered, higher loss factors due occur at small dynamic strains [41,42], which are typical of higher beam-like modes with local deformations [43]. This is also a likely reason why higher modal loss factors could be expected in the CFRP nanocomposite beams undergoing the 3rd bending mode. The significance between the pristine and 0.1 phr panels at the third mode can be also due to the lower fiber volume fraction, which involves a greater resin content and consequently greater CNT content. As shown in Table 4, the 0, 0.2 and 0.5 phr have fiber volume fractions between 49% and 53% while the fiber volume fraction of the 0.1 phr is 43%.

## 5. Conclusions

Nano-epoxies with well-dispersed CNTs were successfully fabricated with a new manufacturing technique, and SEM and AFM imaging were used to investigate the dispersion. The tensile and compressive behaviours of pristine resin and CNT-resin with various weight loads of MWCNTs were investigated to identify statistically significant correlations between the output mechanical properties and the composition of the composites. The addition of small phrs of MWCNTs produced a considerable increase in the traction Young’s modulus and in the compressive strength of the resin. The results match well the theoretical predictions. Comparative vibration tests have confirmed that the MWCNTs epoxy resins can contribute to the vibration damping properties of CFRP-doped with the MWCNTs dispersed with the new technique. This confirmed that MWCNT is not only a highly promising reinforcement for epoxy materials, but also that viable, uniform and large-scale dispersion can be achieved by mechanical techniques without surfactants or diluents.

## Figures and Tables

**Table 1:** Mechanical characteristics of commercially available epoxy resins and CNT-enhanced epoxy in open literature. S stands for sonication, M for mechanical. The mechanical properties are all related to tensile tests.

Name	CNT	Method	wt %	AR (×10^3^)	Young’s Modulus [GPa]		Strength [MPa]		Reference	Note
					Mean	Std	Mean	Std		
DEGBA Epoxy			0.1							
EP-502, Polymer Gvulot	MW	S	0.34	0.4	1.6	0.34	5.7	12.6	[13]	
EP-502, Polymer Gvulot	MW	S	0.34	0.3	1.8	0.64	54	14.9	[13]	Well dispersed
L135i, MGS Kunstharzprodukte	MW	M	0.1	3.3	2.8	0.08	63	0.5	[5]	
L135i, MGS Kunstharzprodukte	MW	M	0.3	3.3	2.8	0.11	63	0.26	[5]	
L135i, MGS Kunstharzprodukte	MW	M	0.5	3.3	2.6	0.03	61	0.38	[5]	Voids detected
DGEBA Epoxy	DW-H2	M	0.1	3.6	3.5	0.22	64	1.6	[16]	
Bisphenol A-epichlorhydrine	MW	methanol	1		0.2		3		[15]	
Bisphenol A-epichlorhydrine	MW	methanol	4		0.5		6		[15]	
Araldite GY 251	SW	S	1		2.3		40		[18]	
Araldite GY 252	F-SW	S	1		2.8		51		[18]	
SX 10 EVO					2.9		65			
Araldite LY 1564					2.5		80			
Super Sap 100					2.5		62			
C System 10 10 CFS							53			
Epon 828 + Epicure 3223					2.8		75			High viscosity

**Table 2:** Tensile tests data.

phr		*σ*		*ε*_yp_		*ε*		Young’s Modulus	
		MPa	*p* *	%	*p*	%	*p*	GPa	*p*
0.00%	Average	73.96		4.14		4.76		3.3	
	std	1.32		0.13		0.19		0.37	
	C.V.	1.78		3.24		4.01		11.32	
0.10%	Average	73.93	0.9820 ns	4.07	0.8419 ns	4.69	0.9373 ns	3.64	0.24 ns
	std	1.72		0.64		1.34		0.39	
	C.V.	2.33		15.76		28.43		10.65	
0.20%	Average	72.09	0.3255 ns	3.19	0.0013 ***	3.27	0.0002 ***	3.89	0.03 *
	std	2.28		0.25		0.27		0.2	
	C.V.	3.16		7.73		8.3		5.11	

Two-tailed *t*-test results comparing each CNTs load with the pristine resin. ns: not significant; * p < 0.05; *** *p* < 0.001.

**Table 3:** Compressive tests results.

phr		*σ*		*ε*_yp_		Young’s Modulus		Young’s Modulus	
		MPa	*p* *	%	*p*	GPa (0.5–0.7%)	*p*	GPa (0.5–2%)	*p*
0.00%	Average	94.86		4.9		2.84		2.897	
	std	4.65		0.36		0.15		0.31	
	C.V.	4.9		7.39		5.25		10.78	
0.10%	Average	96.62	0.5781 ns	3.91	0.0524	2.9	0.6707 ns	3.135	0.33 ns
	std	3.91		0.71		0.18		0.34	
	C.V.	4.05		18.19		6.09		10.75	
0.20%	Average	106.27	0.0050 ***	3.8	0.0623	2.96	0.601 ns	3.028	0.66 ns
	std	3.5		0.69		0.4		0.48	
	C.V.	3.3		18.03		13.66		15.94	
0.50%	Average	113.74	0.0004 ***	6.48	0.206	3.22	0.0311 *	3.156	0.24 ns
	std	3.38		2.08		0.18		0.26	
	C.V.	2.97		32.16		5.44		8.32	

Two-tailed *t*-test results comparing each CNTs load with the pristine resin. ns: not significant; * *p* < 0.05; *** *p* < 0.001.

**Table 4:** Vibration tests results.

CNT		Mode I			Mode II			Mode III			Fiber vol. Fraction
phr		*f* [Hz]	*η*	*p* *	*f* [Hz]	*η*	*p*	*f* [Hz]	*η*	*p*	
0.00%	Mean	40.51	0.063		107.36	0.009		271.39	0.021		49.31%
	std	0.45	0		1.38	0		0.7	0.01		0.004
	C.V.	1.12	4.83		1.28	26.83		0.26	36.48		0.9
0.10%	Mean		0.067	0.235 ns	120.06	0.007	0.263 ns	298.53	0.043	0.006 **	43.44%
	std	40.74	0.01		1.92	0		4.5	0		0.01
	C.V.	0.32	7.52		1.6	17.22		1.51	8.9		2.17
0.20%	Mean	0.78	0.063	0.945 ns	108.76	0.01	0.816ns	277.79	0.032	0.064 ns	49.03%
	std		0.01		3.95	0		6.65	0		0.03
	C.V.	40.43	18.85		3.63	39.38		2.4	15.05		5.22
0.50%	Mean	0.93	0.081	0.611 ns	107.29	0.053	0.0003 ***	253.78	0.044	0.0053 **	52.97%
	std	2.3	0.09		3.5	0.02		3.43	0.01		0
	C.V.		106.74		3.27	30.98		1.35	29.06		0

Two-tailed *t*-test results comparing each CNTs load with the pristine resin. ns: not significant; * p < 0.05; ** *p* < 0.01; *** *p* < 0.001.
